# Construct validity of self-reported and interview-guided administration methods of the Danish version of the post-COVID−19 functional Status scale

**DOI:** 10.3389/fresc.2025.1690892

**Published:** 2025-11-10

**Authors:** Lotte Sørensen, Jane Agergaard, Trine Brøns Nielsen, Berit Schiøttz-Christensen, Cecilia Hee Laursen, Steffen Leth, Claus Vinther Nielsen, Lisa Gregersen Oestergaard

**Affiliations:** 1 Department of Physiotherapy and Occupational Therapy, Aarhus University Hospital, Aarhus, Denmark; 2 Department of Infectious Diseases, Aarhus University Hospital, Aarhus, Denmark; 3 Department of Clinical Medicine, Aarhus University, Aarhus, Denmark; 4 Department of Public Health, Aarhus University, Aarhus, Denmark; 5 DEFACTUM, Aarhus, Central Denmark Region, Denmark; 6 Research Unit of General Practice, Department of Public Health, University of Southern Denmark, Odense, Denmark; 7 Department of Social Medicine and Rehabilitation, Gødstrup Hospital, Herning, Denmark; 8 Department of Internal Medicine and Rehabilitation, Gødstrup Hospital, Herning, Denmark

**Keywords:** construct validity, functional limitations, long-Covid, post-COVID-19 functional status scale, quality of life, sick leave

## Abstract

**Introduction:**

The Post-COVID-19 Functional Status (PCFS) scale was quickly adopted into COVID-19 research and clinical practice worldwide to monitor functional status and recovery. The scale has been translated into Danish, and three different administration methods have been employed. However, clinicians have expressed concerns about the scale's ability to capture work-related functional limitations. Therefore, the purpose of this study was to evaluate the construct validity of three different administration methods of the Danish version of the PCFS scale.

**Methods:**

This cross-sectional study included patients with long COVID who completed three versions of the PCFS scale: a questionnaire-based version, a flowchart-based version, and an interview-based version. The construct validity was evaluated following the Consensus-based Standards for the selection of health Measurement Instruments (COSMIN) guidelines by testing predefined hypotheses that compared the PCFS scale with sick leave and EuroQoL Five-dimensions Five level (EQ-5D-5l).

**Results:**

A total of 437 patients, with a mean age 48 years, 75% female, and 59% on sick leave, were included in this study. Statistically significant differences between the three administration methods were found. Of the 234 patients on sick leave, only 50%-54% had a PCFS grade ≥3 which was below our predefined hypothesis. Furthermore, correlations between the PCFS scale and EQ-5D-5l was lower than hypothesized.

**Conclusion:**

None of the three administration methods effectively captured work-related functional limitations associated with being on part-time or full-time sick leave. Additionally, correlations with quality of life were lower than expected. Overall, the construct validity of the PCFS scale was only partially supported.

## Introduction

Long-term symptoms after infection with the SARS-CoV-2 virus, called Long COVID, are a complex and heterogenic syndrome with symptoms such as fatigue, impaired cognition, dyspnea, muscle and joint pain, muscle exhaustion, and loss of smell and taste (1–3). These symptoms often affect activities of daily living, alter work tasks, result in long-term sick leave, and generally contribute to functional limitations (4–7). Furthermore, the presence of long COVID symptoms is associated with reduced health-related quality of life, even among individuals who were not hospitalized during the acute phase of the infection (8, 9). The majority of patients with long COVID present with rehabilitation needs, and an essential prerequisite for identifying these needs is the assessment of functional status (4, 10). Early in the pandemic, the Post-COVID-19 Functional Status (PCFS) scale was introduced (11) as a slightly adapted version of the Post-Venous thromboembolism Functional Status scale (12, 13). The PCFS scale was rapidly incorporated into COVID-19 research and clinical practice to monitor functional status and recovery and is now available in more than 25 languages (14). The PCFS scale is an ordinal scale ranging from 0 (no functional limitations) to 4 (severe functional limitation) or 5 (death). The scale can be completed either self-reported by the patient using a flowchart and a questionnaire or assessed through a structured interview with a health care professional or trained interviewer. In case of doubt between two grades, the highest grade with the most limitations should be chosen (11).

Several studies have examined the measurement properties of various translated versions of the PCFS scale in patients across various settings, e.g., hospital wards, outpatient clinics, and rehabilitation centres, at different time points since the initial SARS-CoV-2 infection. The studies generally found adequate face validity, construct validity, concurrent validity and reliability (15–19). Individuals with a PCFS score of 2 or higher were found to have an increased number and intensity of symptoms and impairment in work and usual activities (16). In addition, the scale captures the health-related quality of life with the strongest correlations found with the EQ-5D-5l domain “usual activities” (16). Furthermore, a survey conducted among research groups demonstrated that the PCFS scale was perceived as highly user-friendly and was utilized in both research and clinical settings. Additionally, the survey revealed that all scale components, including the patient-reported questionnaire, the flowchart, and the structured interview, were used (20).

The PCFS scale has been translated into Danish and is used in clinical practice and research. However, clinicians in a Danish outpatient Long COVID clinic raised concerns about whether the scale accurately captures work-related functional limitations, particularly in relation to sick leave. In our view, grade 3 of the PCFS scale, “*Usual duties/activities at home or at work have been structurally modified (reduced) due to symptoms, pain, depression, or anxiety,”* represents the most appropriate classification for patients who are on partial or full sick leave. Nevertheless, in practice these patients were frequently assigned a lower grade.

Thus, the purpose of this study stemmed from clinical observations. Furthermore, various administration methods have been employed, and the measurement properties of the Danish version of the scale in general, and those of the different administration methods, remain unclear.

Therefore, the aim of this study was to compare three different administration methods of the Danish PCFS scale: self-reported based on questionnaire, self-reported based on flowchart, and based on interview. Furthermore, the study aimed to evaluate the construct validity of these three administration methods of the PCFS scale.

## Methods

The construct validity was evaluated following the Consensus-based Standards for the selection of health Measurement Instruments (COSMIN) guideline by testing the degree to which the scores of a health-related patient-reported outcome instrument are consistent with predefined hypotheses (21, 22). In this study, the hypotheses addressed the association between the PCFS scale and sick leave, as well as health-related quality of life. Reporting adhered to the COSMIN reporting guideline for studies on measurement properties of patient-reported outcome measures (23).

### Design, settings and participants

This cross-sectional study included patients with persistent symptoms following COVID-19. The diagnosis of COVID-19 was based on a positive polymerase chain reaction (PCR) or antigen test for most patients, while for a few patients, it was based on a clinical evaluation performed by a doctor. Patients were referred by their general practitioner to the outpatient Long COVID clinic, Department of Infectious Diseases, Aarhus University Hospital and diagnosed with long COVID after being examined by a medical doctor at the hospital. In addition, the majority of these patients were examined by an occupational therapist, Department of Physiotherapy and Occupational Therapy, Aarhus University Hospital, using anamnesis and the Canadian Occupational Performance Measure (COPM) to evaluate instrumental activity of daily living, participation in social roles, and rehabilitation needs. Inclusion criteria were a physician-confirmed diagnosis of long COVID and provision of informed consent to participate in the study. Exclusion criteria were lack of completion of all three versions of the PCFS scale. However, for the sensitivity analysis, patients who had completed only the interview-based version of the PCFS scale were included. Patient recruitment took place between February 2021 and January 2023.

### Study procedures

As part of the clinical routine at the Long COVID clinic, patients received a package of electronic questionnaires on their first visit, and 6, 12, and 48 weeks after that. The PCFS scale was included in this package and completed both based on flowchart and on questionnaire (24).

During the clinical visit with the occupational therapist, functional status was assessed using the interview version of the PCFS scale. The occupational therapist guided the patient with questions incorporating the questionnaire and the flowchart (11). In case of doubt regarding a score, the highest score was selected. The visit to the occupational therapist was carried out approximately four to six weeks after the visit at the Long COVID clinic. Therefore, the self-reported PCFS scale, assessed six weeks after the first visit to the Long COVID clinic, was used for comparison in this study.

Patients who completed both the questionnaire-based version, the flowchart-based version, and the interview-based version were included. The fifth grade of the PCFS scale (death) can be used in clinical trials but was left out in this study using self-reported or interview-based versions.

In addition to the PCFS scale, sociodemographic data (age, gender, time since infection, work status/sick leave, educational level, living with spouse/partner, children living at home) was collected through a patient-reported questionnaire. Educational level was categorized as low (primary, lower secondary, or upper secondary), medium (vocational training or short-cycle higher education), or high (bachelor or master).

### Comparison instrument - EQ-5D

Health-related quality of life (HR-QoL) was assessed using the generic EQ-5D-5l questionnaire (25). The instrument consists of a descriptive system that assesses five dimensions: mobility, self-care, usual activities, pain/discomfort, and depression/anxiety (25). These health states reported by patients were weighted into utility indexes using the Danish value set. Utility indexes range from −0.757 to 1.0; a value of 1.0 corresponds to full health, 0 corresponds to death, and negative values correspond to health status considered to be worse than death (26). The Spanish version of the EQ-5D-5l has shown good construct validity and internal consistency, and excellent reliability in patients with long COVID (27).

To assess construct validity, the following primary and secondary hypotheses were tested for each of the three methods of administrating the PCFS scale:

### Primary hypothesis

At least 80% of the patients reporting sick leave (part-time or full-time) had a PCFS score of 3 or higher.

### Secondary hypotheses

The correlation between the EQ-5D-5l total score and the PCFS scale was 0.30-0.50.

The correlation between the EQ-5D-5l domain “usual activities” and the PCFS scale was ≥ 0.50.

The correlation between the EQ-5D-5l domain “mobility” and the PCFS scale was 0.30-0.50.

The correlation between the EQ-5D-5l domain 'self-care' and the PCFS scale was 0.30-0.50.

The correlation between the EQ-5D-5l domain “pain/discomfort” and the PCFS scale was 0.30-0.50.

The correlation between the EQ-5D-5l domain “anxiety/depression” and the PCFS scale was <0.30.

The primary hypothesis was based on the assumption that if a patient reported part-time or full-time sick leave, this should correspond to a PCFS score of ≥3, indicating at least moderate limitations where activities at home or at work have been structurally modified or reduced, as described in the manual (11). This rationale was chosen because sick leave, by definition, reflects a reduction or modification of normal work duties due to health-related limitations, and grade 3 was therefore considered the most appropriate cut-off. To ensure a clinically meaningful threshold, we predefined 80% as the expected proportion, acknowledging that a small minority might be on sick leave for reasons not fully captured by the PCFS scale.

The secondary hypotheses were based on the assumptions that the EQ-5D-5l domain “usual activities” measures a similar construct, the total score and the domains “mobility”, 'self-care', and “pain/discomfort” a related but dissimilar construct, and the domain “anxiety/depression” an unrelated construct.

The construct validity of the PCFS scale was considered acceptable if the primary hypothesis was accepted. The validity was further supported if ≥75% of the secondary hypotheses were accepted.

A minimum of 100 participants were included, as this sample size is considered very good in studies on construct validity.

### Analysis

Sociodemographic data were analysed using descriptive statistics and are presented as means and standard deviation, median and interquartile range (IQR), or absolute and relative frequencies, as appropriate.

The differences between administration methods were tested using a chi-square test. The proportion of patients reporting sick leave with a PCFS score of 3 or higher was reported. The correlations between the PCFS score and the EQ-5D-5l total score, as well as the EQ-5D-5l domains, were analysed using Spearman's rank correlation coefficients with 1,000-repetition bootstrap confidence intervals.

All data were administered through Research Electronic Data Capture (REDCap) (28). Patients were included after giving written informed consent after receiving oral and written information.

## Ethics approval

This study was conducted in accordance with the principles outlined in the Declaration of Helsinki. Approval was sought from the Regional Committee on Biomedical Research Ethics, Central Denmark Region. Following a thorough evaluation, the committee determined that further approval was not necessary (no. 316/1-10-72-181-20). The study was registered in the Central Denmark Region's research database (no. 1-16-02-655-20 and 1-16-02-4-21), and all data were stored and managed in compliance with the General Data Protection Regulation (GDPR). Informed consent was obtained from all participants included in the study. Anonymity and confidentiality were maintained throughout the research process.

## Results

**Figure 1:**
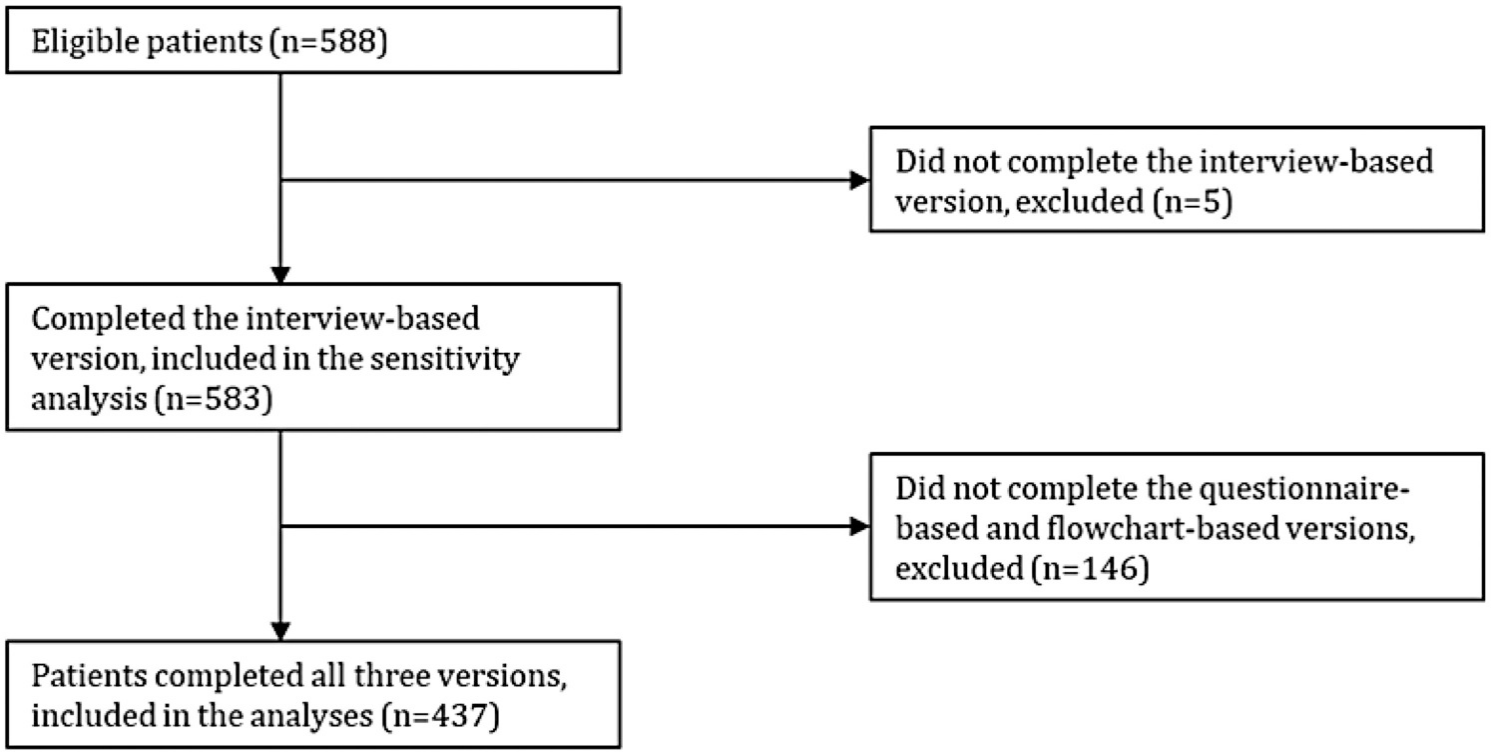
Flowchart of patient inclusion.

During the study period, 588 patients were seen by both a medical doctor at the Long COVID clinic and an occupational therapist. Of these, five patients (0.9%) did not complete the interview-based version and were excluded from the study. A total of 583 patients completed the interview-based version and were included in the sensitivity analysis of the primary hypothesis, and 437 patients (75%) completed all three versions and were included in the final analyses (Figure 1).

Patients included had a mean age of 47.9 years and 75% were female. The patients were assessed with the PCFS scale at a median of approximately eight months after the acute infection. The majority were on sick leave (58.8%) and had a high educational level (54.5%).

Compared with patients who completed all versions of the PCFS scale, those who did not complete the questionnaire-based and the flowchart-based versions were tested longer after the acute infection, had a lower educational level and a larger proportion had been hospitalized. These patients did not complete the EQ-5D-5l either (Table 1).

**Table 1:** Patient characteristics.

Variable	Completed all versions of the PCFS scale, *n* = 437	Not completed the questionnaire-based and flowchart-based versions, *n* = 146	*P*-value
Age, years, mean (SD)	47.9 (11.7)	44.7 (14.0)	0.057
Gender, *n* (%)			
male	111 (25.4)	48 (32.9)	0.079
female	326 (74.6)	98 (67.1)	
Time since infection, days, median (IQR)	243 (162;351)	283 (180;406)	0.045
Hospitalization, *n* (%)			
yes	36 (8.3)	21 (14.4)	0.031
no	400 (91.7)	125 (85.6)	
Work status, *n* (%)			
working/studying same hours as before	150 (37.7)	51 (38.6)	0.832
sick leave	234 (58.8)	75 (56.8)	
unemployed	14 (3.5)	6 (4.6)	
missing	39 (8.9)	14 (9.6)	
Educational level, *n* (%)			
low	53 (12.1)	29 (19.9)	0.030
medium	135 (30.9)	49 (33.6)	
high	238 (54.5)	62 (42.5)	
missing	11 (2.5)	6 (4.1)	
Living with spouse/partner, *n* (%)			
yes	328 (75.2)	100 (68.5)	0.110
no	108 (24.8)	46 (31.5)	
		
Children living at home, *n* (%)			
yes	220 (50.3)	66 (45.2)	0.282
no	217 (49.7)	80 (54.8)	
Mental Fatigue Scale score, mean (SD)	18.2 (5.3)	18.3 (5.5)	0.727
EQ-5D-5l, median (IQR)			
EQ-5D-5l Index	0.794 (0.652;0.873)		
Mobility	0 (0;0.041)		
Self-care	0 (0;0)		
Usual activity	0.040 (0.033;0.139)		
Pain/discomfort	0.094 (0.048;0.094)		
Depression/anxiety	0 (0;0.072)		

EQ-5D-5l, EuroQol 5 Dimension 5 level.

The Mental Fatigue Scale score range from 0 to 45 (29). The EQ-5D-5l utility index range from −0.757 to 1.0 (26).

Using the interview-based version of the PCFS scale, no patients were assessed with grade 0 on the scale. However, 1.4% of the patients were assessed with grade 0 using the questionnaire-based version, and 6.6% of the patients using the flowchart-based version. Grade 4 was used most frequently in the flowchart-based version, while grade 2 was used most frequently in the interview-based version (Table 2).

**Table 2:** Distribution of PCFS scores using three different administration methods, *n* = 437.

PCFS score	Based on structured interview	Self-reported based on questionnaire	Self-reported based on flowchart
Mean, sd	2.39 (0.59)	2.32 (0.70)	2.25 (0.89)
Grade 0, *n* (%)	0 (0.0)	6 (1.4)	29 (6.6)
Grade 1	14 (3.2)	32 (7.3)	26 (6.0)
Grade 2	251 (57.4)	222 (50.8)	208 (47.6)
Grade 3	161 (36.8)	169 (38.7)	155 (35.5)
Grade 4	11 (2.5)	8 (1.8)	19 (4.4)

The interview-based version and the questionnaire-based version of the PCFS scale differed statistically significantly (*p* < 0.001). Of the 437 included patients, 286, 65% (95% CI 61;70) reported the same grade using both administration methods (Table 3).

**Table 3:** Comparison of the interview-based and the questionnaire-based version of the PCFS scale, *n* = 437.

PCFS interview	PCFS self-reported questionnaire					
PCFS score	Grade 0	Grade 1	Grade 2	Grade 3	Grade 4	Total
Grade 0	0	0	0	0	0	0
Grade 1	0	7	6	0	1	14
Grade 2	5	21	166	59	0	251
Grade 3	1	4	48	107	1	161
Grade 4	0	0	2	3	6	11
Total	6	32	222	169	8	437

Gray shading indicates identical grades in the two compared versions of the PCFS scale.

The interview-based version and the flowchart-based version differed statistically significantly (*p* < 0.001). Of the 437 included patients, 269, 62% (95% CI 57;66) reported the same grade using both administration methods (Table 4).

**Table 4:** Comparison of the interview-based and the flowchart-based version of the PCFS scale, *n* = 437.

PCFS interview	PCFS self-reported flowchart					
PCFS score	Grade 0	Grade 1	Grade 2	Grade 3	Grade 4	Total
Grade 0	0	0	0	0	0	0
Grade 1	4	4	6	0	0	14
Grade 2	22	19	155	46	9	251
Grade 3	3	3	45	105	5	161
Grade 4	0	0	2	4	5	11
Total	29	26	208	155	19	437

Gray shading indicates identical grades in the two compared versions of the PCFS scale.

The questionnaire-based version and the flowchart-based version of the PCFS scale differed statistically significantly (*p* < 0.001). Of the 437 included patients, 268, 61% (57;66) reported the same grade using both administration methods (Table 5).

**Table 5:** Comparison of the questionnaire-based and the flowchart-based version of the PCFS scale, *n* = 437.

PCFS self-reported questionnaire	PCFS self-reported flowchart					
PCFS score	Grade 0	Grade 1	Grade 2	Grade 3	Grade 4	Total
Grade 0	3	0	0	2	1	6
Grade 1	7	12	10	2	1	32
Grade 2	17	10	144	42	9	222
Grade 3	2	4	52	106	5	169
Grade 4	0	0	2	3	3	8
Total	29	26	208	155	19	437

Gray shading indicates identical grades in the two compared versions of the PCFS scale.

Testing the primary hypothesis revealed that none of the versions of the PCFS scale demonstrated acceptable construct validity. Of 234 patients reporting sick leave (part-time or full-time), 127, 54% (95% CI 48;61) had a PCFS grade of 3 or higher using the interview-based version, 125, 53% (95% CI 47;60) using the questionnaire-based version, and 116, 50% (95% CI 43;56) using the flowchart-based version. A sensitivity analysis including all patients completing the interview-based version (*n* = 583) showed similar results; of the 309 patients reporting sick leave, 158, 51% (95% CI 45;57) had a PCFS grade of 3 or higher. Furthermore, the construct validity was not supported by the secondary hypotheses, as less than 75% were accepted (33% for the interview-based and the flowchart-based version and 50% for the questionnaire version) (Table 6).

**Table 6:** Spearman rank correlations between the PCFS scale and results of testing the secondary hypotheses, *n* = 437.

EQ-5D-5l	Based on structured interview		Self-reported based on questionnaire		Self-reported based on flowchart	
	*r*	Hypothesis	*r*	Hypothesis	*r*	Hypothesis
EQ-5D-5l _total score_	−0.35 (−0.44; −0.27)	+	−0.44 (−0.52; −0.37)	+	−0.33 (−0.42; −0.24)	+
EQ-5D-5l _usual activity_	0.50 (0.42;0.57)	-	0.56 (0.49;0.62)	+	0.43 (0.35;0.52)	-
EQ-5D-5l _mobility_	0.22 (0.13;0,31)	-	0.25 (0.16;0,34)	-	0.16 (0.07;0,26)	-
EQ-5D-5l _self−care_	0.12 (0.11;0.29)	-	0.23 (0.13;0.32)	-	0.18 (0.09;0.27)	-
EQ-5D-5l _pain/discomfort_	0.15 (0.05;0,24)	-	0.28 (0.19;0,36)	-	0.17 (0.07;0.27)	-
EQ-5D-5l _anxiety/depression_	0.13 (0.03;0.23)	+	0.12 (0.03;0,21)	+	0.06 (−0.03;0.16)	+

+, hypothesis accepted; -, hypothesis not accepted.

## Discussion

At the Long COVID Clinic, a large cohort of patients with a confirmed diagnosis of long COVID was established. Using this cohort, several studies were conducted employing different administration methods of the Danish version of the PCFS scale, enabling us to compare and evaluate the construct validity of the interview-based, questionnaire-based, and flowchart-based versions of the PCFS scale.

The main findings of this study revealed a statistically significant difference between the three administration methods, indicating that these methods cannot be used interchangeably and highlighting the importance of reporting how the scale is assessed. Furthermore, only between 50% and 54% of patients on sick leave had a PCFS grade of 3 or higher across the three methods, which did not meet our primary hypothesis that at least 80% of the patients reporting sick leave (either part-time or full-time) would achieve a PCFS score of 3 or higher. This debatable assumption highlights potential weaknesses in the PCFS scale's ability to capture functional limitations in patients on sick leave. We had anticipated that patients on sick leave (part-time or full-time) would correspond to grade 3, which is described as “Usual duties/activities at home or at work have been structurally modified (reduced) due to symptoms, pain, depression or anxiety”.

The limited ability of the PCFS scale to capture work-related functional limitations may reflect the fluctuating and multidimensional nature of long COVID symptoms, including post-exertional malaise, fatigue, and cognitive difficulties that affect work ability even when basic activities are maintained (7, 30). These features differ from the more stable recovery patterns in the post-venous thromboembolism population for which the scale was designed.Since our primary hypothesis was not supported, concerns arise regarding the PCFS scale's ability to capture the full extent of work-related functional limitations. As far as we know, this approach has not been used in other studies. Furthermore, the construct validity was not supported by our secondary hypothesis, which evaluated the correlations between the PCFS scale and EQ-5D-5l. The low correlations found in this study indicate that functional limitations and HR-QoL are distinct aspects of a person's life for patients with long COVID.

Other studies have evaluated the measurement properties of the PCFS scale in various languages. Generally, it has been concluded that the PCFS scale has acceptable measurement properties and is recommended for use across clinical settings and research (15–19, 31). However, the administration method used was often poorly described, and none of these studies evaluated predefined hypotheses as recommended by the COSMIN guidelines. Furthermore, only one study assessed the construct validity of the PCFS scale in comparison to work ability or sick leave (16). The study of Machado et al. found that patients classified as grade 2 or higher presented a gradual increase in the number and intensity of symptoms and impairment in work and usual activities, as well as a reduction in HR-QoL (16). Impairment in work and usual activities was measured with the Work Productivity and Activity Impairment questionnaire (WPAI), from which four main domains can be assessed: absenteeism, presenteeism, work impairment due to health, activity impairment due to health. Furthermore, HR-QoL was assessed with EQ-5D-5l and like in our study, the EQ-5D-5l domain “usual activity” showed the strongest correlation with the PCFS scale compared to the other domains, although all correlations were higher than found in our study. The study by Machado et al. recruited patients through Facebook and a web-based registration of lung symptoms, and an online survey was used as administration method (16). Compared to the study of Sacristán-Galisteo et al., we found lower correlations between the PCFS scale and the EQ-5D-5l total score, *r* = 0.33-0.44 vs. *r* = 0.83 (19). The observed lower correlations likely reflect the differing focus of the two instruments. The EQ-5D-5l measures general health-related quality of life, whereas the PCFS scale captures condition-specific functional limitations, explaining its greater relevance to post-COVID-19 functioning. These findings highlight the need to revalidate existing tools for new clinical populations and to develop measures that more accurately reflect the functional impact of long COVID. A key limitation of the study is that not all text in the PCFS scale manual, especially the structured interview questions, was translated into Danish. This may have introduced variability in how the occupational therapists conducted the interviews, potentially affecting the validity of this administration method. The face-to-face examination conducted by the occupational therapist included anamnesis, assessment using the COPM, and a dialogue with the patient regarding basic activities of daily living, instrumental activities of daily living, and participation in usual social roles. Therefore, we anticipated that the interview-based assessment of the PCFS scale would closely adhere to the manual. Surprisingly, the results revealed that the occupational therapist did not succeed in capturing the importance of sick leave. Nonetheless, systematic application of the interview questions might have improved consistency, particularly regarding work-related functional limitations. The translation process of the PCFS scale from English to Danish is believed to have been conducted in accordance with international guidelines; however, no additional information or considerations have been published.

Additionally, a significant portion of the patients (25%) did not complete the self-reported versions of the PCFS scale. However, bias is less likely, as no significant difference in the PCFS score was observed between those completing all versions and those completing only the interview-based version. Furthermore, including all patients interviewed by the occupational therapist did not change the agreement between the interview-based version and sick leave.

The two self-reported versions of the PCFS scale were assessed concurrently, approximately six weeks after the first visit to the outpatient clinic and within a few weeks from the consultation with the occupational therapist. However, the agreement between the two self-reported versions was lower than that observed when compared to the interview-based version, indicating that the different time points likely did not influence the result.

The post-COVID-19 functional status is intended to be assessed at the time of discharge, shortly after discharge (e.g., 4 and 8 weeks), and again 6 months post-discharge (11). In the present study, fewer than 10% of the patients had been hospitalized, and patients were assessed at a median of approximately eight months after the acute infection. As a result, this study did not adhere to the standardization of measurement regarding time window and population. However, long COVID symptoms often occur among non-hospitalized patients. Moreover, the patients included in this study had experienced ongoing long COVID symptoms for several months at the time of assessment, and their referral to the clinic reflected the lack of spontaneous improvement in these symptoms.

This study, as many others using the PCFS scale during the pandemic, was conducted under pressing circumstances (14). However, we believe that this evaluation of the psychometric properties of the scale in a clinical setting that integrates practice and research significantly enhances the interpretation of these findings.

Based on results from this study, future application of the PCFS scale should focus on providing clear instructions to healthcare professionals for using the interview-based version and to patients when using the self-reported version. Return-to-work evaluations should not be based exclusively on PCFS grade, but instead incorporate a comprehensive appraisal of functional capacity and symptom persistence. Furthermore, revalidation of the underlying structure of the PCFS scale is recommended, including a thorough factor analysis to examine its dimensionality and ensure that the scale accurately reflects the intended construct of post-COVID functional status.

## Conclusion

This study identified significant differences between the three administration methods of the Danish PCFS scale: self-reported via questionnaire, self-reported via flowchart, and scored by an occupational therapist through interview. None of the methods effectively captured work-related functional limitations associated with being on part-time or full-time sick leave. Additionally, correlations with HR-QoL measured by EQ-5D-5l were lower than hypothesised. Overall, we conclude that the construct validity of the PCFS scale was only partially supported, suggesting limitations in its ability to reflect work-related functioning and quality of life across administration methods.

## Data Availability

The datasets presented in this article are not readily available because the data that support the findings of this study are not openly available due to reasons of sensitivity. The datasets used and/or analysed during the current study are available from the corresponding author on reasonable request. Requests to access the datasets should be directed to Lotte Sørensen, lotsoere@rm.dk.

## References

[1] O'Mahoney LL Routen A Gillies C Ekezie W Welford A Zhang A The prevalence and long-term health effects of long COVID among hospitalised and non-hospitalised populations: a systematic review and meta-analysis. EClinicalMedicine. (2023) 55:101762. 10.1016/j.eclinm.2022.101762 36474804 PMC9714474

[2] Michelen M Manoharan L Elkheir N Cheng V Dagens A Hastie C Characterising long COVID: a living systematic review. BMJ Glob Health. (2021) 6(9):e005427. 10.1136/bmjgh-2021-005427 34580069 PMC8478580

[3] Lopez-Leon S Wegman-Ostrosky T Perelman C Sepulveda R Rebolledo PA Cuapio A More than 50 long-term effects of COVID-19: a systematic review and meta-analysis. Sci Rep. (2021) 11(1):16144. 10.1038/s41598-021-95565-8 34373540 PMC8352980

[4] Nielsen TB Leth S Pedersen M Harbo HD Nielsen CV Laursen CH Mental fatigue, activities of daily living, sick leave and functional Status among patients with long COVID: a cross-sectional study. Int J Environ Res Public Health. (2022) 19(22):14739. 10.3390/ijerph192214739 36429458 PMC9690484

[5] Chasco EE Dukes K Jones D Comellas AP Hoffman RM Garg A. Brain fog and fatigue following COVID-19 infection: an exploratory study of patient experiences of long COVID. Int J Environ Res Public Health. (2022) 19(23):15499. 10.3390/ijerph192315499 36497573 PMC9737348

[6] Vanichkachorn G Newcomb R Cowl CT Murad MH Breeher L Miller S Post-COVID-19 syndrome (long haul syndrome): description of a multidisciplinary clinic at mayo clinic and characteristics of the initial patient cohort. Mayo Clin Proc. (2021) 96(7):1782–91. 10.1016/j.mayocp.2021.04.024 34218857 PMC8112396

[7] Davis HE Assaf GS McCorkell L Wei H Low RJ Re'em Y Characterizing long COVID in an international cohort: 7 months of symptoms and their impact. EClinicalMedicine. (2021) 38:101019. 10.1016/j.eclinm.2021.101019 34308300 PMC8280690

[8] Mercier K Piché J Rioux-Perreault C Lemaire-Paquette S Piché A. A longitudinal prospective cohort study of health-related quality of life assessment in outpatient adults with post-COVID-19 conditions. J Assoc Med Microbiol Infect Dis Can. (2024) 8(4):309–18. 10.3138/jammi-2023-0010 38250617 PMC10797766

[9] Carlile O Briggs A Henderson AD Butler-Cole BFC Tazare J Tomlinson LA Impact of long COVID on health-related quality-of-life: an OpenSAFELY population cohort study using patient-reported outcome measures (OpenPROMPT). Lancet Reg Health Eur. (2024) 40:100908. 10.1016/j.lanepe.2024.100908 38689605 PMC11059448

[10] Chuang HJ Lin CW Hsiao MY Wang TG Liang HW. Long COVID and rehabilitation. J Formos Med Assoc. (2024) 123(Suppl 1):S61–s9. 10.1016/j.jfma.2023.03.022 37061399 PMC10101546

[11] Klok FA Boon G Barco S Endres M Geelhoed JJM Knauss S The post-COVID-19 functional Status scale: a tool to measure functional status over time after COVID-19. Eur Respir J. (2020) 56(1):2001494. 10.1183/13993003.01494-2020 32398306 PMC7236834

[12] Klok FA Barco S Siegerink B. Measuring functional limitations after venous thromboembolism: a call to action. Thromb Res. (2019) 178:59–62. 10.1016/j.thromres.2019.04.003 30980999

[13] Boon G Barco S Bertoletti L Ghanima W Huisman MV Kahn SR Measuring functional limitations after venous thromboembolism: optimization of the post-VTE functional Status (PVFS) scale. Thromb Res. (2020) 190:45–51. 10.1016/j.thromres.2020.03.020 32298840

[14] de Jong CMM Le YNJ Boon G Barco S Klok FA Siegerink B. Eight lessons from 2 years of use of the post-COVID-19 functional Status scale. Eur Respir J. (2023) 61(5):2300416. 10.1183/13993003.00416-2023 37080570 PMC10151454

[15] de Facio CA Guimarães FS da Cruz AGT Bomfim RF Miranda S Viana DR Post-COVID-19 functional status scale: cross-cultural adaptation and measurement properties of the Brazilian Portuguese version. Braz J Phys Ther. (2023) 27(3):100503. 10.1016/j.bjpt.2023.100503 37201307 PMC10126223

[16] Machado FVC Meys R Delbressine JM Vaes AW Goërtz YMJ van Herck M Construct validity of the post-COVID-19 functional Status scale in adult subjects with COVID-19. Health Qual Life Outcomes. (2021) 19(1):40. 10.1186/s12955-021-01691-2 33536042 PMC7856622

[17] Tsekoura M Fousekis K Billis E Dionyssiotis Y Tsepis E. Cross-cultural adaptation of the Greek version of post-COVID-19 functional Status scale: assessment of non-hospitalised post-COVID-19 survivors. Eur J Transl Myol. (2023) 33(2):11328. 10.4081/ejtm.2023.11328 37345497 PMC10388593

[18] Çalık Kütükcü E Çakmak A Kınacı E Uyaroğlu OA Yağlı NV Sain Güven G Reliability and validity of the turkish version of post-COVID-19 functional Status scale. Turk J Med Sci. (2021) 51(5):2304–10. 10.3906/sag-2105-125 34392673 PMC8742502

[19] Sacristán-Galisteo C Del Corral T Ríos-León M Martín-Casas P Plaza-Manzano G López-de-Uralde-Villanueva I. Construct validity of the Spanish version of the post-COVID-19 functional Status scale and validation of the web-based form in COVID-19 survivors. PLoS One. (2022) 17(6):e0269274. 10.1371/journal.pone.0269274 35648770 PMC9159614

[20] de Jong CMM Boon G Le YNJ Barco S Siegerink B Klok FA. The post-venous thromboembolism functional Status scale: from call to action to application in research, extension to COVID-19 patients, and its use in clinical practice. Semin Thromb Hemost. (2023) 49(7):764–73. 10.1055/s-0043-1764467 36940713

[21] Mokkink LB Prinsen CA Bouter LM Vet HC Terwee CB. The COnsensus-based standards for the selection of health measurement INstruments (COSMIN) and how to select an outcome measurement instrument. Braz J Phys Ther. (2016) 20(2):105–13. 10.1590/bjpt-rbf.2014.0143 26786084 PMC4900032

[22] de Vet HCW Terwee CB Mokkink LB Knol DL. Measurement in Medicine: A Practical Guide. Cambridge: Cambridge University Press (2011).

[23] Gagnier JJ Lai J Mokkink LB Terwee CB. COSMIN Reporting guideline for studies on measurement properties of patient-reported outcome measures. Qual Life Res. (2021) 30(8):2197–218. 10.1007/s11136-021-02822-4 33818733

[24] Agergaard J Ullahammer WM Gunst JD Østergaard L Schiøttz-Christensen B. Characteristics of a Danish post-COVID cohort referred for examination due to persistent symptoms six months after mild acute COVID-19. J Clin Med. (2022) 11(24):7338. 10.3390/jcm11247338 36555954 PMC9783804

[25] Feng YS Kohlmann T Janssen MF Buchholz I. Psychometric properties of the EQ-5D-5l: a systematic review of the literature. Qual Life Res. (2021) 30(3):647–73. 10.1007/s11136-020-02688-y 33284428 PMC7952346

[26] Jensen CE Sørensen SS Gudex C Jensen MB Pedersen KM Ehlers LH. The Danish EQ-5D-5l value set: a hybrid model using cTTO and DCE data. Appl Health Econ Health Policy. (2021) 19(4):579–91. 10.1007/s40258-021-00639-3 33527304 PMC8270796

[27] Fernández-de-Las-Peñas C Rodríguez-Jiménez J Moro-López-Menchero P Cancela-Cilleruelo I Pardo-Hernández A Hernández-Barrera V Psychometric properties of the Spanish version of the EuroQol-5D-5l in previously hospitalized COVID-19 survivors with long COVID. Sci Rep. (2022) 12(1):12605. 10.1038/s41598-022-17033-1 35871259 PMC9307967

[28] Harris PA Taylor R Minor BL Elliott V Fernandez M O'Neal L The REDCap consortium: building an international community of software platform partners. J Biomed Inform. (2019) 95:103208. 10.1016/j.jbi.2019.103208 31078660 PMC7254481

[29] Johansson B Rönnbäck L. Evaluation of the mental fatigue scale and its relation to cognitive and emotional functioning after traumatic brain injury or stroke. Int J Phys Med Rehabil. (2014) 2(01):1000182. 10.4172/2329-9096.1000182

[30] O'Brien KK Brown DA McDuff K St Clair-Sullivan N Solomon P Chan Carusone S Conceptualising the episodic nature of disability among adults living with long COVID: a qualitative study. BMJ Glob Health. (2023) 8(3):e011276. 10.1136/bmjgh-2022-011276 PMC9979585 36863719

[31] Moreno-Torres LA Ventura-Alfaro CE. Validation of the post-COVID-19 functional Status scale into Mexican-Spanish. J Rehabil Med Clin Commun. (2021) 4:1000070. 10.2340/20030711-1000070 34659654 PMC8505751

